# Manufacture accuracy and geometric discrepancy of direct laser sintering versus cast cobalt chromium bars in mandibular implant retained overdenture: an in-vitro comparative study

**DOI:** 10.1186/s12903-026-09358-4

**Published:** 2026-07-24

**Authors:** Ehab A. Farrag, Mohammed E. El-Sayed, Ahmed A. Moselhy, Kawkb M. El-Tamimi

**Affiliations:** 1 Department of Removable Prosthodontics, Faculty of Dentistry, Suez Canal University, Ismailia, Egypt; 2 Department of Removable Prosthodontics, Military Medical Academy, Cairo, Egypt

**Keywords:** Cobalt-Chromium, Dental Implant, Manufacture accuracy, Geometric discrepancy, 3D printing, Laser sintering, Overdenture

## Abstract

**Background:**

This in vitro study aimed to compare and analysis degree of Manufacture accuracy and geometric discrepancy measurements of Cobalt-Chromium (C0-Cr) bar joint attachment supporting a mandibular complete overdenture fabricated directly by Direct Laser Sintering (DMLS) and indirectly fabricated by conventional casting of 3D printed resin pattern using Co-Cr alloys with the same reference design.

**Methods:**

A total of twelve bar joint attachment for two implant-retained mandibular overdentures were fabricated through additive manufacturing process indirectly by using 3D printed resin pattern and directly by using DMLS (n = six for each group). All the sample specimens of metal bars were scanned and superimposed with the reference STL reference design by using the Geomagic Control X software program.

**Results:**

There was no significant statistical difference in manufacture accuracy and geometric discrepancy in the bars fabricated by DMLS compared to indirectly 3D-printed conventional Co-Cr bars in mandibular implant retained overdenture. The Casted group has a mean of 0.1128 ± 0.006, while the DMLS group has a mean of 0.1206 ± 0.01. There was no significant difference between the groups, Regarding Internal Discrepancy, the Casted group shows a mean of 0.1659 ± 0.118, and the DMLS group has a mean of 0.1678 ± 0.035. The research indicates no significant difference between the groups for this variable.

**Conclusion:**

No significant difference in Manufacture accuracy and geometric discrepancy of direct laser sintering versus cast 3D printed resin pattern of Co-Cr bars in mandibular implant retained complete overdenture.

## Introduction

Edentulism adversely impacts physiological function, esthetics, social interactions, and psychological well-being [1]. Conventional complete dentures are frequently associated with inadequate retention and stability, mucosal ulceration, pain, and patient discomfort, which may subsequently compromise mastication, speech, nutrition, and overall oral function [2].

Implant-retained overdentures (IOD) represent a predictable and effective treatment modality for edentulous patients, offering superior retention, stability, and functional performance compared with conventional complete dentures [3]. Implant overdenture bar attachments may be manufactured either conventionally through wax pattern fabrication followed by investment and casting using the lost-wax technique, or digitally using CAD/CAM technology [4]. Co–Cr alloys are commonly selected due to their favorable mechanical properties, durability, and cost-effectiveness [5].

The scientific basis of this study lies in the accurate fabrication of implant-supported bar attachments for mandibular implant-retained overdentures. These frameworks play a critical role in splinting implants and facilitating the distribution of occlusal forces to the supporting bone [6]. The long-term success of such prostheses largely depends on achieving a passive fit between the bar and the implants, thereby minimizing stress transmission to the implant–bone interface [7].

Dimensional discrepancies between the fabricated bar and implant components may compromise passive fit, generating mechanical strain. Such misfit has been associated with biological and mechanical complications, including peri-implant bone loss, screw loosening, framework fracture, and reduced prosthesis longevity [8].

Conventional (Co–Cr) alloys bar fabrication is commonly performed using the lost-wax casting technique. However, the multiple laboratory procedures involved, including wax pattern fabrication, investing, casting, and finishing, are susceptible to cumulative dimensional inaccuracies resulting from material shrinkage, thermal changes, and operator-dependent variables [9].

Advances in CAD/CAM technologies have introduced DMLS, an additive manufacturing technique in which Co-Cr frameworks are fabricated directly from digital designs through selective melting of metal powder layers [5]. This approach has the potential to reduce manufacturing variability and improve dimensional accuracy by minimizing manual processing steps. Nevertheless, the accuracy of DMLS produced frameworks remains influenced by factors such as laser parameters, thermal gradients, and layer thickness [10].

Gaps in the current literature remain evident, as framework adaptation has been assessed predominantly through two-dimensional evaluation methods, including the Sheffield test, optical microscopy, and cross-sectional scanning electron microscopy (SEM) [11]. These approaches provide limited information regarding the overall three-dimensional accuracy and volumetric distortion of the framework Although these techniques quantify localized misfit, they fail to adequately characterize three-dimensional volumetric distortion, multidirectional deformation, and overall spatial discrepancies throughout the entire geometry of long-span mandibular bars [12]. Consequently, comprehensive full-surface 3D colorimetric comparisons between DMLS and conventionally cast frameworks are still scarce [13].

Furthermore, available evidence is largely derived from in vitro studies employing rigid gypsum casts or dimensionally stable implant analogs [14]. This limits the clinical applicability of the findings, as the behavior of manufacturing inaccuracies under functional intraoral conditions remains insufficiently understood. In particular, the influence of framework misfit in the presence of mandibular flexure, variations in implant angulation, and resilient attachment mechanisms has not been thoroughly investigated [8].

Another unresolved issue concerns the effect of post-processing procedures. DMLS frameworks commonly undergo stress-relief heat treatment and polishing to reduce surface irregularities generated during layer-by-layer fabrication; however, data describing dimensional changes throughout these stages are limited. As a result, the extent to which thermal treatment and finishing procedures contribute to distortion development or dimensional correction remains uncertain [15].

In addition, current research has focused predominantly on two-implant overdenture bars, while evidence regarding longer-span, multi-implant mandibular frameworks remains limited. The association between span length, inter-implant distance, and cumulative dimensional distortion has not been systematically established. Likewise, comparative analyses evaluating thermal deformation in DMLS frameworks versus solidification shrinkage in Co–Cr castings across extended spans are lacking [16]. Therefore, this study was undertaken to evaluate and compare the manufacturing accuracy and geometric discrepancy of DMLS and conventionally Co-Cr bars. By quantifying deviations from the intended design.

Previous literatures have reported inconsistent findings regarding the adaptation accuracy of CAD-CAM and conventional fabrication techniques. Several studies have demonstrated favorable outcomes for additive manufacturing technologies. Castillo-Oyagüe et al. and Hama Suleiman et al. reported that DMLS fabricated copings exhibited smaller marginal discrepancies and superior internal adaptation compared with conventionally cast restorations [17, 18]. Similarly, Stamenković et al. suggested that selective laser melting (SLM) frameworks may offer manufacturing advantages, including reduced technical complexity and improved mechanical properties, although they emphasized the need for further investigation regarding adaptation accuracy and build orientation [19]. More recently, Singla et al. concluded that DMLS is a promising alternative to conventional casting, reporting fewer casting defects, enhanced mechanical properties, and clinically acceptable marginal fit for both single-unit and multi-unit prostheses [20]. 

In contrast, other studies have favored conventional fabrication methods. Katsoulis et al., Park et al., and Nesse et al. found that conventionally cast restorations demonstrated superior marginal adaptation compared with DMLS fabricated counterparts [21–23]. Likewise, Jun Yang and Hainan Li reported no significant precision advantage for CAD-CAM milling over conventional lost-wax casting and observed superior marginal adaptation in restorations produced from handmade conventional wax patterns [24]. Collectively, these conflicting findings indicate that the influence of fabrication technique on restoration adaptation remains inconclusive and may be affected by variations in manufacturing protocols, materials, and assessment methodologies. Therefore, this study was undertaken to evaluate and compare the manufacturing accuracy and geometric discrepancy of DMLS and conventionally cast Co-Cr bars. By quantifying deviations from the intended design,

The null hypothesis (H₀) stated that there would be no statistically significant difference in manufacture accuracy and geometric discrepancy between DMLS fabricated and conventionally casted Co–Cr bars. The alternative hypothesis (H₁) proposed that a statistically significant difference would exist between the two fabrication techniques.

## Materials and methods

### Simulation model preparation and wax cast fabrication

Silicone rubber mold (H90Type High quality blue silicone material model former made in 3/8″ thick material Kilgore International. Inc, U. S) was used to duplicate a gypsum cast into wax cast. Blue lab wax heated to melting temperature then fill the mold and left to harden.

### Implant positioning and placement

A Surveyor (Ney Surveyor, Dentsply, USA) used to locate sites for placing the two implants at least 11 mm length and 3.75 mm in diameter inside the wax cast by mounting the implant to an open tray impression post then using the surveyor to achieve position, centricity, parallelism of the inserted implant in canine regions.

**Fig. 1:**
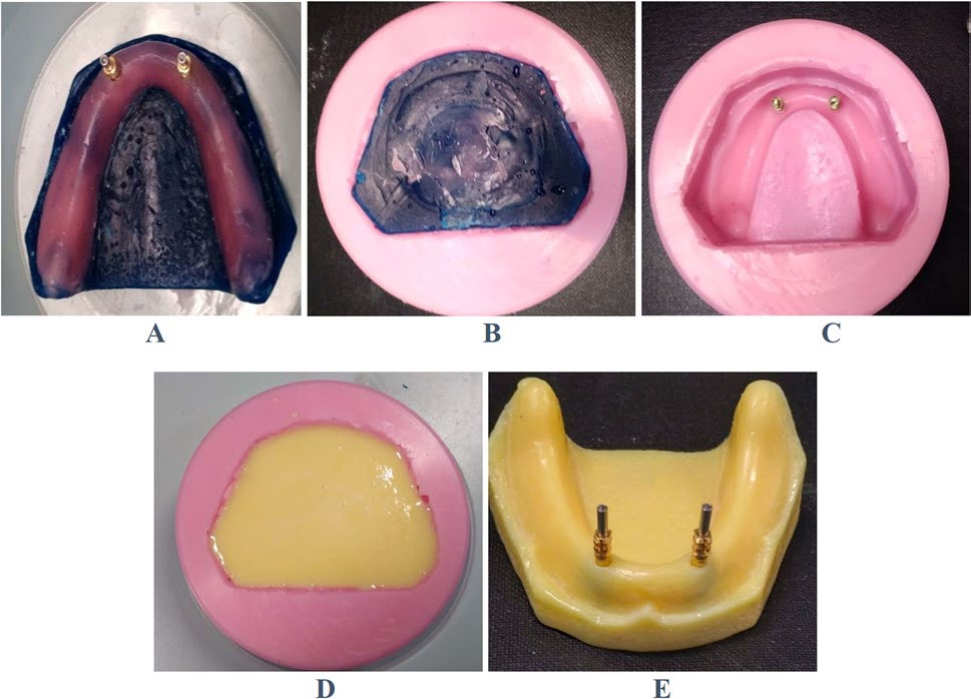
**A** 2 open tray impression post mounted to 2 implant parallel placed in the wax replicas placed in a container. **B** epoxy cast construction duplicating the cast with silicone rubber mold B, rubber modding silicon after softening all the blue wax; **C** implant +impression post in silicon mold after melting the blue wax; **D** final epoxy cast; **E** epoxy cast after removing from the silicon rubber mold

The impression posts then were connected to each other using self-cured acrylic resin to fabricate affixation jigs to prevent movement of the implants during construction of the epoxy cast. The wax cast with the implants then replicated by using rubber silicon material. (Fig. 1A, B) [25]. By melting the wax by using hot water then pouring the rubber mold with epoxy resin material (Kimapoxy 150 3D, CMB international, Egypt) to produce epoxy cast. (Fig. 1C-E).

### Digital model design

The cast was optically 3D scanned with dental desktop structured-light scanner as the cast was fixed to the cast holder which rotated automatically to scan the cast from all directions. Optical scanning by (Medit t700, Seol, South Korea) used to calculate the position of implants and high precision scan adaptors mounted on the implants. So, the visual or digital planning involved taking a scan of the denture and a scan of the cast, and we designed the bar segment visually, aligning it with the position of the clip. It was done this way to ensure it remains entirely within the confines of the acrylic. Multiunit abutment (SGS Switzerland) was inserted into implant platform and scan body (SGS Switzerland) was applied into their sites covering the abutments.

**Fig. 2:**
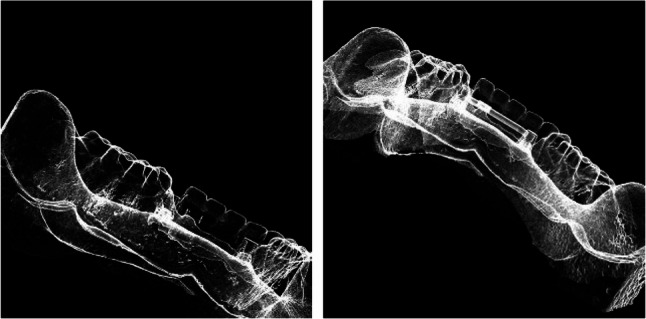
Hader bar fully design on the CAD-CAM program

Scanner Spray (Occlutec scan spray Renfert, Germany), was used to spray the epoxy resin cast prior to scanning for a more accurate scan and the generated 3D model. Finally, the cast was scanned and the design of the bar completed digitally to achieve full digital workflow of the study cast and the bar attachment, the data was exported as Standard Tessellation Language (STL) file (Fig. 2).

### Bar framework grouping and identification

Sample size calculation was performed using G*Power version 3.1.9.2 [26]. The effect size conventions (d) were 1.6(large) based on previous studies [27]. Using alpha (α) level of 0.05 and Beta (β) level of 0.20, i.e., power = 80%; the estimated sample size (n) should be 12 samples and was divided equally for two groups (6 samples each).

All frameworks after digitally designing were numerically marked from numbers 1 to 12 (*n* = 12) and all located into two groups (*n* = 6*/*group) according to the fabrication method and technique.

Group (I (six 3D-printed resin pattern conventionally Co-Cr bars (from No.one up to No. six).

Group (II) six DMLS Co-Cr bar designs (from No. seven up to No. 12) represented direct metal laser sintering bar group. Each group was evaluated for 2D comparisons, 3D comparisons and analysis were performed.

### 3D-printed resin bar pattern fabrication

3D printed resin bar patterns were fabricated by using a 3D printer (Creality Halot, China) liquid crystal display based on stereolithography (SLA) system coupled with synergistic biocompatible photosensitive resin material in liquid form depending on STL file data. Liquid crystal display (LCD) system of the printing machine used a digital light projector to flash a single image of an entire layer all at ones; this made it faster than SLA.

A photosensitive liquid resin layer is selectively polymerized through ultraviolet (UV) laser exposure, resulting in hardening according to the predefined pattern. Object fabrication is achieved by sequentially curing successive resin layers using projected light, with each cross-sectional layer polymerized simultaneously in a layer-by-layer process [28].

The STL file of the bar framework design was 3D printed from liquid resin using stereography. The printer created 3D printed resin bar framework by selectively curing a polymer resin layer by layer in range between 25 and 100 micron,15s exposure time and one second break with building angle 45 using an ultraviolet laser beam [29].

The resin vat base consists of a Fluorinated Ethylene Propylene (FEP) film that permits light transmission, while the light source is positioned beneath the vat [30].

The build platform is lowered into a vat of liquid resin (UV green resin for 3D printers EPAX, China), a UV light source projects the first layer of the model onto the surface of the resin, and the resin hardens in the shape of the first layer, The build platform is raised slightly, and the process is repeated for each layer of the model. It followed top-to bottom orientation. The default settings were two second cure times at 60% brightness at the beginning.

Resin printers typically contain a single moving component driven by one motor and lack dedicated heating or cooling systems, apart from a small electronic cooling fan [31]. The standard layer thickness of the EPAX printer (Creality Halot, China) is 50 μm, which provides approximately fourfold higher resolution compared to the standard FDM layer thickness [32].

Following fabrication of the resin pattern framework, it was meticulously detached from the build platform using the provided plastic spatula. Subsequent post-processing involved multiple curing stages, including immersion in ethyl alcohol to eliminate residual uncured resin, followed by final polymerization in a UV curing chamber to achieve complete hardening [33].

### Casting of 3D-printed resin patterns

**Fig. 3:**
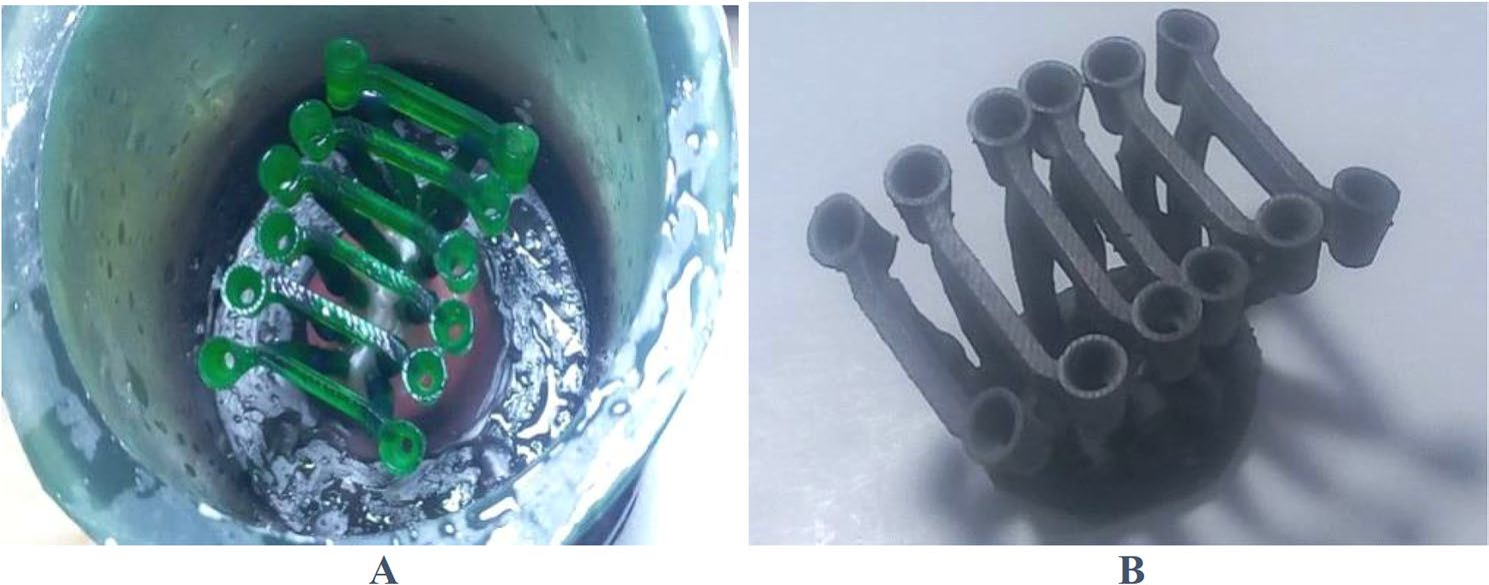
Casting of printed resin

First, the pattern is 3D printed in a resin material, which is then used as a template for the metal investment (Exavest, Shanghai, China) and casting. Even though this method’s design process is streamlined using CAD, it still requires labor-intensive casting and investing methods, and the restoration was subsequently re-evaluated for fit on the cast. A tree sprue system was then attached, and the components were invested using a phosphate-bonded precision investment material. (Fig. 3A) [34].

The pattern was invested directly with phosphate bonded investment under pressure with manufacturer’s instructions and cast in conventional technique as the investment ring is placed in burnout furnace where the printing resin (UV green resin for 3D printers) was burned out at 900 °c for 1–8 s until all resin particles were vaporized leaving clean empty mold for casting. An electrometric centrifugal induction casting machine used for casting. Co-Cr alloy powder (Starbond easy pulver 30 – powder type 5 - S&S Schefyner Gmb, Germany) was used to cast the bars at 1200°cas (Fig. 3B) [35].

Element components of Co-Cr molten alloy are shown (Table 1).

**Table 1:** Element components of cast Co-Cr alloy

Co	Cr	Mo	Mn	C, Si
65%	28%	5%	1%	1%

### Fabrication of direct metal laser-sintered cobalt–chromium bar frameworks

The second approach involves directly fabricating the frameworks using metal additive manufacturing. A 3D metal printing machine VULCANTECH VM120 was used to complete the printing of the bar profile using Co-Cr alloy powder as the printing material.

**Fig. 4:**
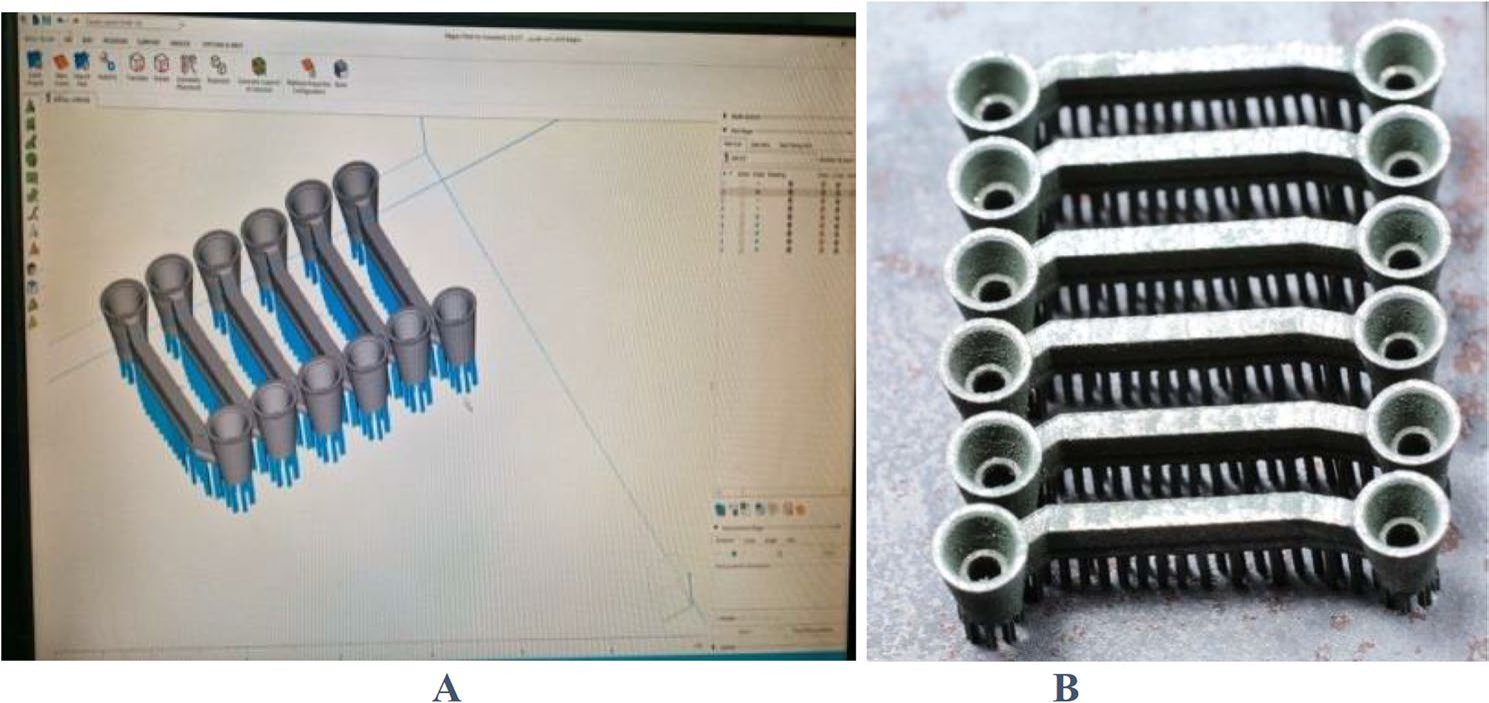
**A** metal printed bars virtually constructed, **B** bars after printing

The original STL file of the bar design was imported into Magic print for Vulcan to generate a 70% support structure, producing a revised STL file with support arms oriented toward the exterior surface (Fig. 4A). This file was transferred to a 3D printing system employing a high-power fiber laser (197–200 W) to selectively melt and fuse Co–Cr alloy powder (particle size 10–30 μm) in 25 μm layers. Printing was performed with a 45° build orientation [36]. A 45° build orientation was adopted, incorporating a linear support configuration supplemented with horizontal reinforcement structures. Processing parameters included a laser spot diameter of 0.08–0.10 mm, a sintering speed of 1100–1200 mm/s, and a layer thickness of 20 μm [37].

Element components of printed powder are shown in (Table 2).

**Table 2:** Element components of printed powder

Co	Cr	W	Si	Fe	Mn
61%	27.5%	8.5%	1.6%	< 0.5%	< 0.1%

Upon completion of printing, the platform was removed from the printer, and the samples were separated by cutting the support arms using an air-turbine handpiece. All six samples underwent this procedure and were subsequently sintered to full density in the designated sintering furnaces (Fig. 4B) [38].

### Finishing and polishing of bar frameworks

All frameworks underwent standardized finishing and polishing procedures. Following detachment from the build platform, support structures were removed, support tips were separated, and excess metal was eliminated. Surface refinement was achieved by sandblasting with 50 μm aluminum oxide (Al₂O₃) particles at 0.5 MPa using a sandblasting unit (Renfert-GmbH Basic Classic Sandblaster, Germany) to remove residual roughness and excess material. Final polishing was performed using fine rubber polishers, brushes, and polishing paste.

In the present study, the support structures were intentionally designed and positioned away from the fitting surfaces that were evaluated for geometric discrepancy. This design strategy ensured that the critical measurement areas were not directly affected by the support removal procedure. The removal of support structures was carefully standardized to minimize any potential variability: All specimens were processed by a single experienced operator, eliminating inter-operator variability. A consistent protocol was followed using the same air-turbine handpiece, cutting burs, and operating conditions Importantly, the support structures were intentionally designed and positioned away from the fitting/measurement surfaces of the specimens. Therefore, their removal did not involve or affect the critical geometrical regions used in the discrepancy analysis. Support removal was limited strictly to the support-arm junctions, with no additional finishing or polishing procedures performed. Each specimen was visually inspected after support removal to ensure that no unintended surface alteration occurred prior to scanning.

### Scanning and digitalization

Prior to digitization, all framework surfaces were coated with scan spray (Occlutec, Germany) to produce a uniform matte finish. Each specimen was scanned using a high-resolution laboratory scanner under controlled ambient temperature conditions. The frameworks were positioned with the occlusal surface oriented superiorly, then re-scanned after a 180° rotation. This protocol was applied to all 12 frameworks, generating an individual raw scan file for each specimen.

The obtained datasets were converted to OBJ format for analysis. STL files were subsequently imported into Geomagic software, where deviations of the geometric centers of each framework from the reference STL design were quantified using Geomagic Control X (Version 2018). Final fabricated restorations were assessed through combined visual and manual inspection for comparative evaluation.

### Manufacturing accuracy and geometrical discrepancy

Manufacture accuracy and geometric discrepancies of the metal bars were assessed via 3D analysis, comparing the reference design with both directly printed and indirectly fabricated samples. The internal surfaces contacting the multiunit abutment were left untreated.

**Fig. 5:**
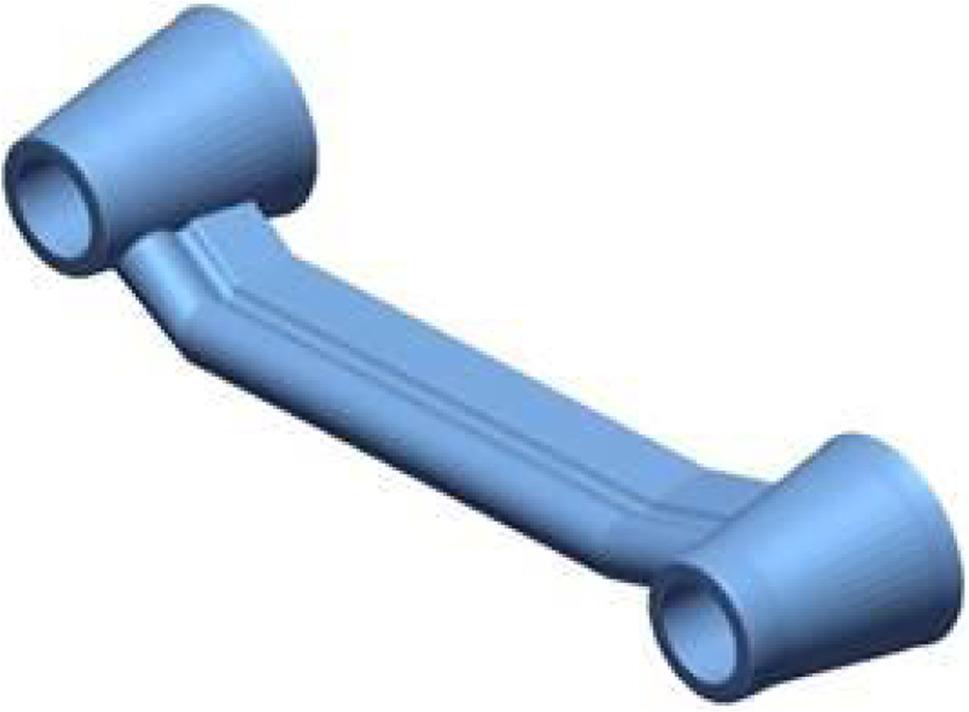
Reference STL design accuracy samples

Each scanned framework STL file was aligned with the corresponding sectioned region of the original design STL. Accuracy was assessed using Geomagic Control X by superimposing the scanned STL onto the original STL and applying a best-fit algorithm within a 3D inspection software environment (Fig. 5).

**Fig. 6:**
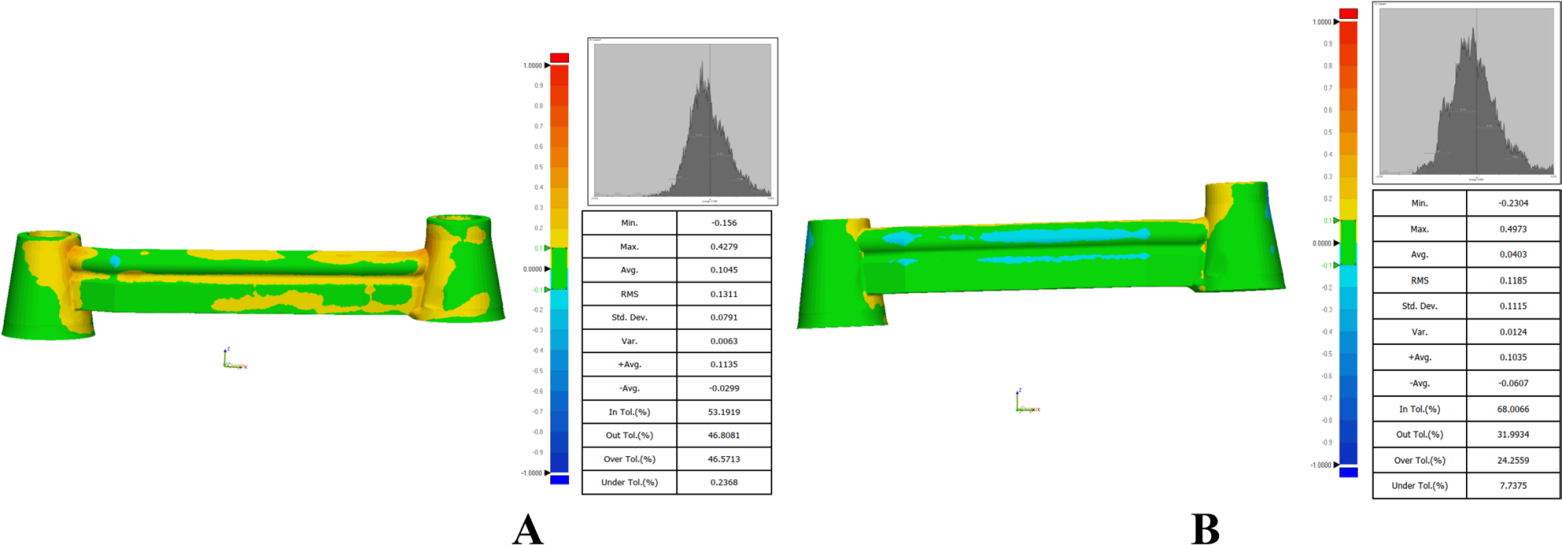
**A**, **B** Framework accuracy was assessed through comparative 3D color-map analysis

Geometrical discrepancies of the internal framework surfaces were analyzed using comparative 3D analysis, and overall framework accuracy was evaluated through color-mapped comparative 3D analysis (Figs. 6A, B).

**Fig. 7:**
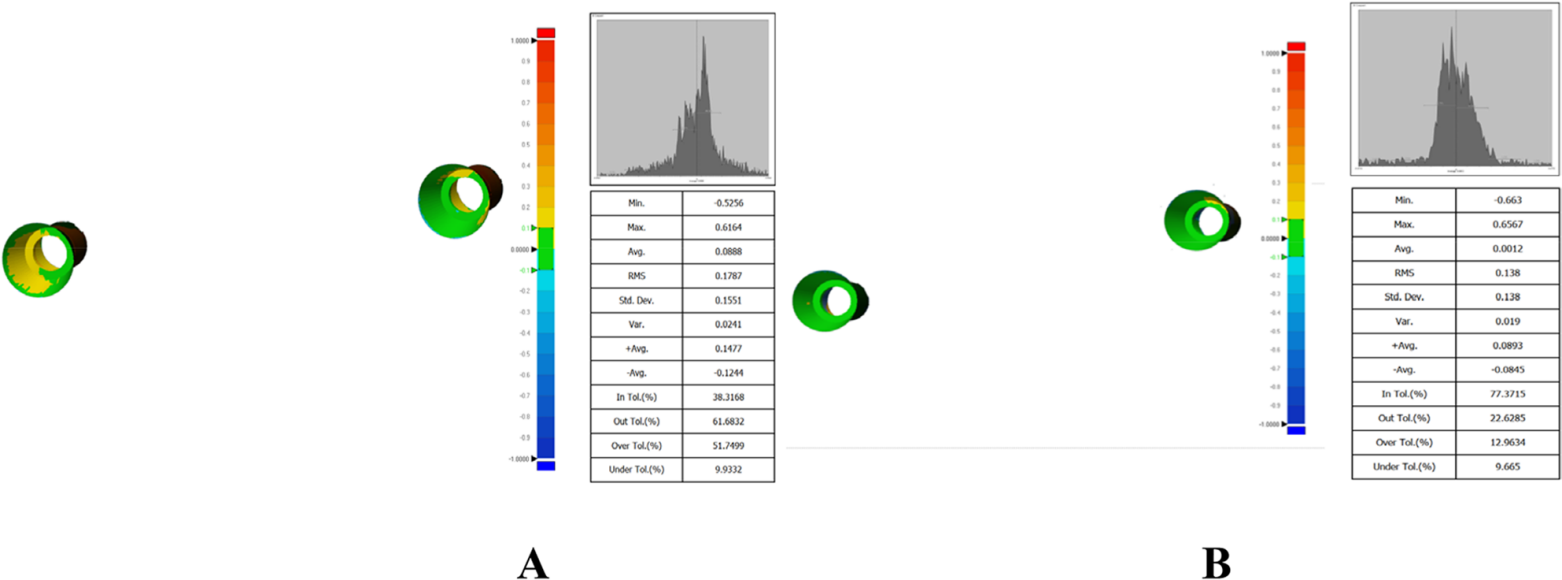
**A**, **B** Measurement of Internal Discrepancy from the Original STL Design

Internal discrepancy was determined by segmenting the original STL design and comparing it with the internal fit of each sample, followed by overlay with the corresponding sectioned region of the initial STL (Figs. 7A, B).

**Fig. 8:**
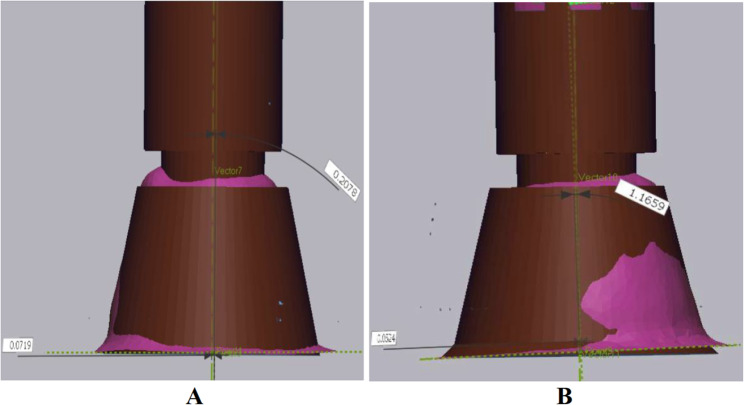
**A**, **B** Measurement of horizontal discrepancy from the original STL design

**Fig. 9:**
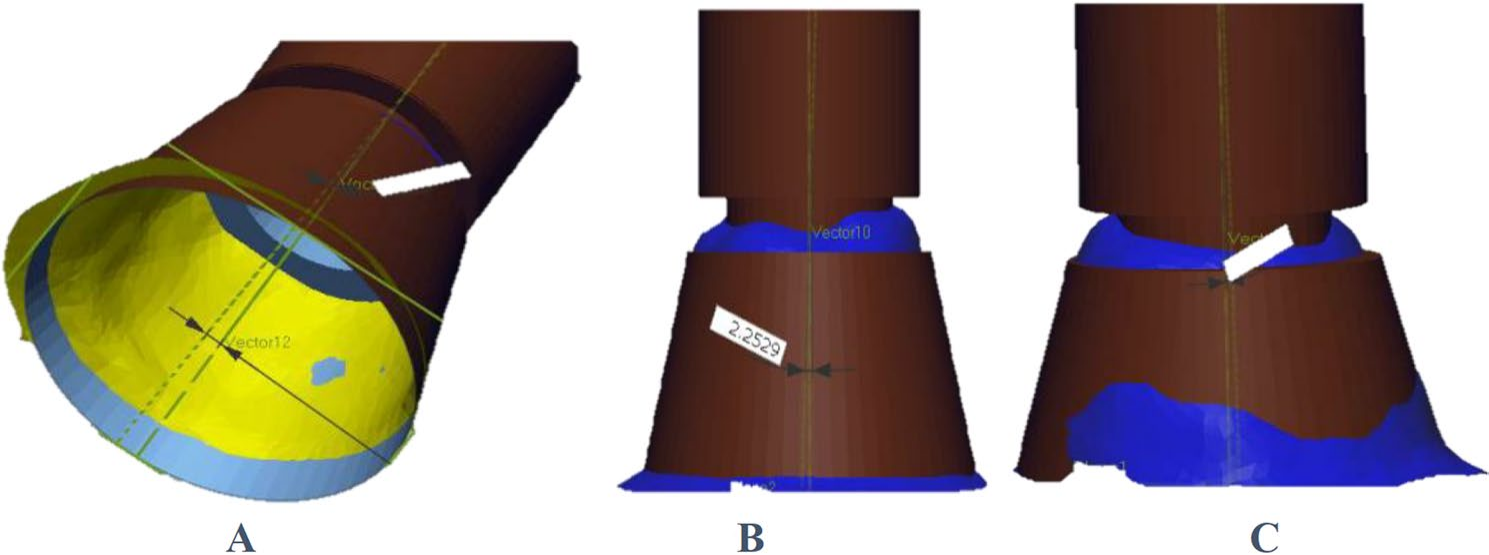
**A** **B**, **C** Angular deviation from the original STL design

The differences in horizontal discrepancy and angular discrepancy on the right side and on left side measured then compared with corresponding side on the original STL design (Figs. 8A-B, and 9A-C).

## Results

**Fig. 10:**
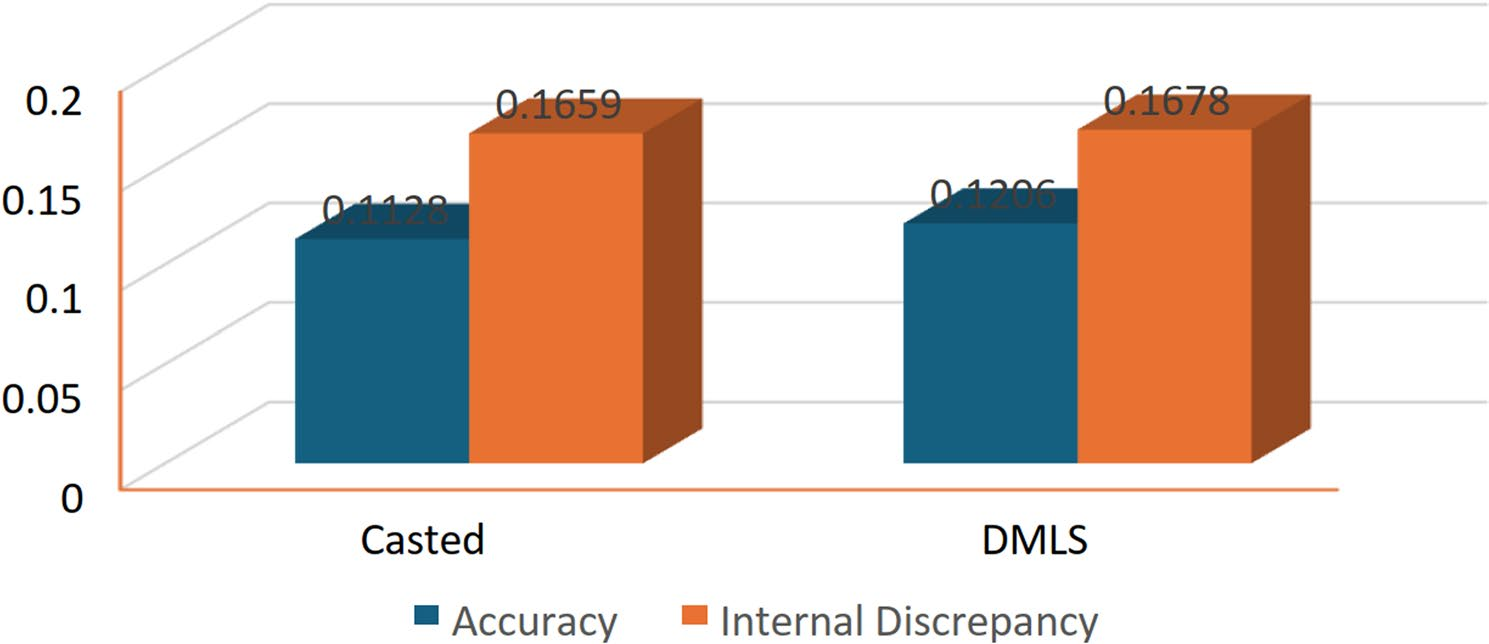
Comparison between the two groups for accuracy and internal discrepancy

Table 3, comparison between the two groups for Accuracy and Internal Discrepancy for Accuracy, the Casted group has a mean of 0.1128 ± 0.006, while the DMLS group has a mean of 0.1206 ± 0.01. The independent t-test resulted in a p-value of 0.386, indicating no significant difference between the groups. (Fig. 10)

**Table 3:** Comparison between the two groups for Accuracy and Internal Discrepancy

Variables	Groups				Independ. T-test	*p*-value
	Casted	SD	DMLS	SD		
Accuracy	0.1128 a	0.006	0.1206 a	0.01	0.074	0.386 (ns)
Internal Discrepancy	0.1659 a	0.118	0.1678 a	0.035	0.5	0.241 (ns)

Ns: mean no significant difference at *P* < 0.05 Test used: independent samples T test

Regarding Internal Discrepancy, the Casted group shows a mean of 0.1659 ± 0.118, and the DMLS group has a mean of 0.1678 ± 0.035. The independent t-test yielded a p-value of 0.241, which also indicates no significant difference between the groups for this variable.

Table 4, for Horizontal Discrepancy on the right side, the Casted group has a mean of 0.117, while the DMLS group has a mean of 0.069. This difference is statistically significant (*p* = 0.030). On the left side, the Casted group has a mean of 0.139, and the DMLS group has a mean of 0.102, with no significant difference between the groups (*p* = 0.204). Right and left side in both groups showed non-significant difference with *P* > 0.05.

**Fig. 11:**
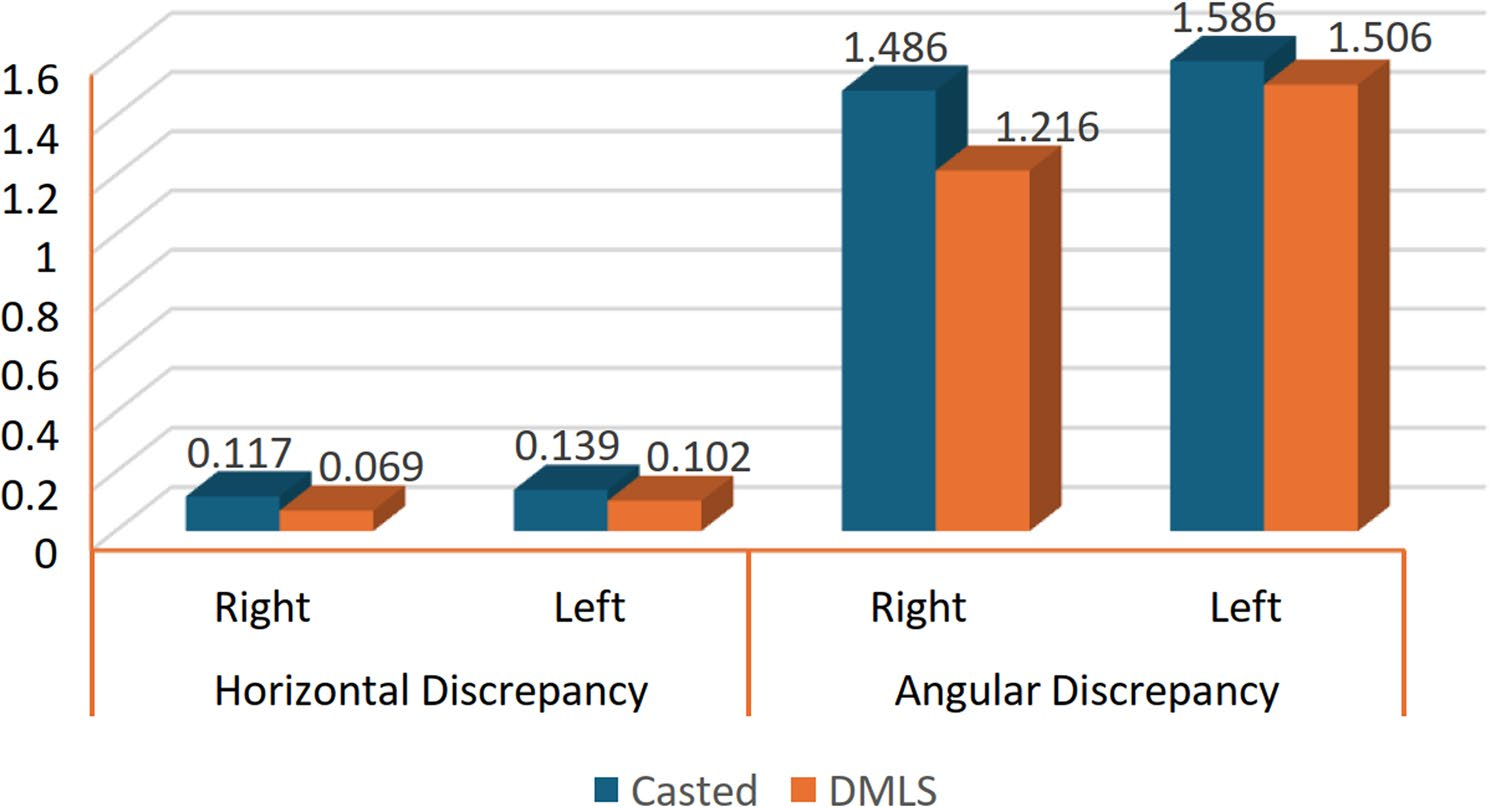
Comparison between the two groups at Horizontal and Angular Discrepancy in right and left sides

Regarding Angular Discrepancy, on the right side, the Casted group shows a mean of 1.486, and the DMLS group has a mean of 1.216. On the left side, the Casted group has a mean of 1.586, and the DMLS group has a mean of 1.506. For both sides, the differences between the groups are not statistically significant (right: *p* = 0.191, left: *p* = 0.434). Right and left side in both groups showed non-significant difference with *P* > 0.05. (Fig. 11).

**Table 4:** Comparison between the two groups at Horizontal and Angular Discrepancy in bright and left sides

Variables	Sides	Groups				*p*-value
		Casted		DMLS		
		Mean	SD	Mean	SD	
Horizontal Discrepancy	Right	0.117 a	0.051	0.069 b	0.02	0.030 *
	Left	0.139a	0.095	0.102	0.047	0.204 ns
	Ind.t test	0.313 ns		0.077 ns		
Angular Discrepancy	Right	1.486a	0.671	1.216a	0.282	0.191 ns
	Left	1.586a	0.847	1.506a	0.742	0.434 ns
	Paired t-test	0.414 ns		0.194 ns		

* mean significant, Ns; non-significant difference at *P* < 0.05

Test used: independent samples T test

## Discussion

Casting techniques for bar implant supported over denture fabrication, while established, have several limitations. These include technique complexity, susceptibility to errors, and the need for high levels of technical skill [39]. An increase in the number of steps involved in the casting procedure heightens the risk of technical errors. The primary aim of casting is to achieve precise metallic reproduction of dental prostheses with optimal manufacture accuracy [40].

The fabrication of an implant-retained overdenture bar assembly encompasses multiple clinical and laboratory procedures, including impression making, master cast production, bar wax-up, spruing and investing, followed by casting and finishing. Each stage carries a risk of distortion, which may compromise the passive fit of the final restoration [41].

DMLS is a CAD/CAM-based technology in which prostheses are digitally designed using specialized software, and the design data are subsequently transmitted to the MLS unit for fabrication. The prosthesis is produced through successive layering of Co–Cr alloy powder with an approximate thickness of 20 μm per layer, where alloy particles are sintered by a high-powered laser in a repetitive process until complete prosthesis formation is achieved [42].

Numerous studies have evaluated DMLS technique and demonstrated its promising potential for dental applications compared with the conventional lost-wax casting (CLW) technique [43].

The introduction of digital impressions using intra-oral optical scanner (IOS) into the fields of fixed and implant prosthodontics aims to aid in achieving this goal while it carries advantages, namely the elimination of tray selection, reduced risks of distortion during impression making, pouring, disinfecting, and shipping to the laboratory, potentially increased patient comfort and acceptance and finally electronic storage as digital information [44].

The passive fit of prosthodontic frameworks is critical to prevent mechanical and biological complications in multiple implant rehabilitation [45]. A lack of passive fit of the definitive prosthesis may cause mechanical (screw loosening or screw fracture and monolithic prosthesis fracture) or biological (mucositis or peri-implantitis) complications [46].

Considering the critical role of the abutment–implant interface in maintaining stability and ensuring the long-term success of implant-supported restorations, thorough assessment of the influence of different manufacturing techniques on internal fit and interface accuracy remains essential [47].

The present in vitro study aimed to evaluate the manufacturing accuracy and geometric discrepancy of Co–Cr bars fabricated using DMLS compared with those produced by conventional casting for mandibular implant-retained overdentures. Based on the results obtained, the null hypothesis (H₀) that there would be no statistically significant difference in manufacturing accuracy and geometric discrepancy between DMLS fabricated and conventionally cast Co–Cr bars were accepted.

Overall, the available evidence remains inconclusive. While additive manufacturing techniques such as DMLS and SLM demonstrate technological and mechanical advantages, their superiority in terms of marginal adaptation and geometric accuracy over conventional lost-wax casting or CAD-CAM milling has not been consistently established. Further standardized comparative studies are required to clarify these discrepancies.

The limitations of this study were the relatively small sample size, future studies with greater sample sizes are required to assess the effect of construction technique (DMSL or conventional) on manufacture accuracy and geometric discrepancy of Co-Cr bars in mandibular implant retained overdenture and later to be applied in-vivo.

## Conclusion

Within the limitations of this in vitro study, it can be concluded that there was no statistically significant difference in manufacturing accuracy and geometric discrepancy between DMLS and conventionally cast Co-Cr bars for mandibular implant-retained overdentures.

## Data Availability

The results/data/figures in this manuscript have not been published elsewhere, nor are they under consideration (from you or one of your Contributing Authors) by another publisher.

## Declarations

During the preparation of this manuscript, the authors used ChatGPT to improve the linguistic quality and grammatical structure of the text.

## Abbreviations

CAD/CAM: Computer-aided design and Computer-aided manufacture
Co-Cr: Cobalt-chromium
DMLS: Direct metal laser sintering technology
FDM: Fused deposition modeling
FEP: Fluorinated ethylene propylene
IODs: Implant-retained overdentures
LCD: Liquid crystal display
MPa: Megapascals
RMS: Root mean square
SLA: Stereolithography
SLM: Selective laser melting
SM: Subtractive manufacturing
STL: Standard Tessellation Language
UV: Ultraviolet
